# Supplementary material for: Estimating the costs of adolescent HIV care visits and an intervention to facilitate transition to adult care in Kenya
Source: PLoS One. 2024 Feb 8;19(2):e0296734. doi: 10.1371/journal.pone.0296734 (PMC10852328; doi:10.1371/journal.pone.0296734)
Supplement: S1 Appendix — (DOCX) [file pone.0296734.s001.docx]

# S1 Appendix. Monte Carlo simulation used to estimate parameter uncertainty.

For each parameter with measured variability (i.e., standard deviation or inter-quantile range) we assumed a prior probability distribution. We simulated several deviates independently for each parameter and followed the process previously described to estimate the costs per session with each deviate. Thus, we obtained a distribution of the cost per session for the control and intervention clinics and estimated the mean and quantile-based 95% interval. We created density plots for the cost of care for both control and intervention settings to generate a comprehensive understanding of the uncertainty in the estimates and differences between intervention and control cost estimates.
