# Supplementary material for: Estimating the costs of adolescent HIV care visits and an intervention to facilitate transition to adult care in Kenya
Source: PLoS One. 2024 Feb 8;19(2):e0296734. doi: 10.1371/journal.pone.0296734 (PMC10852328; doi:10.1371/journal.pone.0296734)
Supplement: S2 Appendix — (DOCX) [file pone.0296734.s002.docx]

# S2 Appendix. Number of records collected on each instrument, by clinic.

| Clinic | Status | Time-Motion | Costing Interview |
| --- | --- | --- | --- |
| AHF | Control | 2 | 0 |
| Gilgil | Control | 2 | 0 |
| Kajiado | Control | 5 | 5 |
| Kangemi | Control | 4 | 4 |
| Kitare | Control | 5 | 5 |
| Nyagoro | Control | 4 | 4 |
| Kitengela | Intervention | 8 | 5 |
| Langalanga | Intervention | 3 | 0 |
| Loitoktok | Intervention | 2 | 0 |
| Makadara | Intervention | 5 | 5 |
| Mathare | Intervention | 2 | 5 |
| Miriu | Intervention | 3 | 3 |
| Rachuonyo | Intervention | 3 | 3 |
