# Supplementary material for: Estimating the costs of adolescent HIV care visits and an intervention to facilitate transition to adult care in Kenya
Source: PLoS One. 2024 Feb 8;19(2):e0296734. doi: 10.1371/journal.pone.0296734 (PMC10852328; doi:10.1371/journal.pone.0296734)
Supplement: S3 Appendix — (DOCX) [file pone.0296734.s003.docx]

# S3 Appendix. Respondents’ self-reported roles and activities by type of facility.

|  | **Control (N=48)** | **Intervention (N=80)** | **Total (N=128)** | **p value** |
| --- | --- | --- | --- | --- |
| **Employment** |  |  |  | < 0.001^1^ |
| Clinical Officer | 9 (18.8%) | 6 (7.5%) | 15 (11.7%) |  |
| Counselor | 24 (50.0%) | 10 (12.5%) | 34 (26.6%) |  |
| Nurse | 2 (4.2%) | 5 (6.2%) | 7 (5.5%) |  |
| Nurse Counselor | 9 (18.8%) | 54 (67.5%) | 63 (49.2%) |  |
| Other Role | 0 (0.0%) | 3 (3.8%) | 3 (2.3%) |  |
| Psychologist | 4 (8.3%) | 2 (2.5%) | 1. (4.7%) |  |

^1^Pearson’s Chi-squared test

*Notes: Twenty HCWs described their role as ‘peer educator’, ‘community health volunteer’, and ‘mentor mother’ which fell under the role of counselor. The remaining record described their job as ‘link desk attendant,’ and was labeled as “other role.”*
