# Supplementary material for: Estimating the costs of adolescent HIV care visits and an intervention to facilitate transition to adult care in Kenya
Source: PLoS One. 2024 Feb 8;19(2):e0296734. doi: 10.1371/journal.pone.0296734 (PMC10852328; doi:10.1371/journal.pone.0296734)
Supplement: S4 Appendix — (DOCX) [file pone.0296734.s004.docx]

# S4 Appendix. Respondents’ reported activities by type of facility.

|  | Control (N=48) | Intervention (N=80) | Total (N=128) | p value |
| --- | --- | --- | --- | --- |
| **Activity** |  |  |  | 0.010^1^ |
| Blood draw in laboratory | 1 (2.1%) | 2 (2.5%) | 3 (2.3%) |  |
| Blood draw in office | 1 (2.1%) | 0 (0.0%) | 1 (0.8%) |  |
| Booklet Review | 0 (0.0%) | 22 (27.5%) | 22 (17.2%) |  |
| Checking-in | 7 (14.6%) | 8 (10.0%) | 15 (11.7%) |  |
| Counseling | 13 (27.1%) | 21 (26.2%) | 34 (26.6%) |  |
| Other Activity | 3 (6.2%) | 1 (1.2%) | 4 (3.1%) |  |
| Overall Assessment | 7 (14.6%) | 8 (10.0%) | 15 (11.7%) |  |
| Prescription dispensing | 7 (14.6%) | 6 (7.5%) | 13 (10.2%) |  |
| Triage | 9 (18.8%) | 12 (15.0%) | 21 (16.4%) |  |

^1^Pearson’s Chi-squared test

*Notes: Fifteen participants listed “other” for the activities they ordinarily conduct. Five participants’ description aligned with counseling, four with overall assessment, and three with prescription dispensing, and the remaining with booking for future appointments. All records were assigned to the corresponding pre-defined activities.*
