# Supplementary material for: Estimating the costs of adolescent HIV care visits and an intervention to facilitate transition to adult care in Kenya
Source: PLoS One. 2024 Feb 8;19(2):e0296734. doi: 10.1371/journal.pone.0296734 (PMC10852328; doi:10.1371/journal.pone.0296734)
Supplement: S5 Appendix — (DOCX) [file pone.0296734.s005.docx]

# S5 Appendix. Time-motion instrument: Estimated times per activity.

| Activity |  | Control (N=50) | Intervention (N=57) | Total (N=107) | p value |
| --- | --- | --- | --- | --- | --- |
| Blood draw in laboratory |  |  |  |  | NaN^1^ |
|  | Mean | 5.00 | 5.00 | 5.00 |  |
|  | SD | NA | NA | 0.00 |  |
|  | N | 1 | 1 | 2 |  |
| Blood draw in office |  |  |  |  | 0.683^1^ |
|  | Mean | 3.00 | 4.00 | 3.50 |  |
|  | SD | 1.41 | 2.83 | 1.91 |  |
|  | N | 2 | 2 | 4 |  |
| Booking |  |  |  |  | 0.468^1^ |
|  | Mean | 5.75 | 12.00 | 8.88 |  |
|  | SD | 5.91 | 15.38 | 11.29 |  |
|  | N | 4 | 4 | 8 |  |
| Booklet Review |  |  |  |  |  |
|  | Mean | NA | 15.00 | 15.00 |  |
|  | SD | NA | 11.54 | 11.54 |  |
|  | N | 0 | 15 | 15 |  |
| Checking-in |  |  |  |  | 0.638^1^ |
|  | Mean | 3.56 | 4.75 | 3.92 |  |
|  | SD | 2.24 | 3.86 | 2.72 |  |
|  | N | 9 | 4 | 13 |  |
| Counseling |  |  |  |  | 0.574^1^ |
|  | Mean | 20.80 | 19.42 | 20.05 |  |
|  | SD | 8.69 | 7.79 | 8.04 |  |
|  | N | 10 | 12 | 22 |  |
| Overall Assessment |  |  |  |  | 0.531^1^ |
|  | Mean | 13.25 | 16.00 | 14.71 |  |
|  | SD | 6.84 | 8.22 | 7.50 |  |
|  | N | 8 | 9 | 17 |  |
| Prescription dispensing |  |  |  |  | 0.107^1^ |
|  | Mean | 5.20 | 3.00 | 4.22 |  |
|  | SD | 2.49 | 1.41 | 2.28 |  |
|  | N | 5 | 4 | 9 |  |
| Triage |  |  |  |  | 0.613^1^ |
|  | Mean | 6.64 | 12.67 | 8.76 |  |
|  | SD | 3.64 | 13.34 | 8.53 |  |
|  | N | 11 | 6 | 17 |  |

^1^Kruskal-Wallis rank sum test
