# Supplementary material for: Estimating the costs of adolescent HIV care visits and an intervention to facilitate transition to adult care in Kenya
Source: PLoS One. 2024 Feb 8;19(2):e0296734. doi: 10.1371/journal.pone.0296734 (PMC10852328; doi:10.1371/journal.pone.0296734)
Supplement: S6 Appendix — (DOCX) [file pone.0296734.s006.docx]

# S6 Appendix. Costing instrument: Estimated times per activity.

| Activity |  | Control (N=20) | Intervention (N=36) | Total (N=56) | p value |
| --- | --- | --- | --- | --- | --- |
| Booklet Review |  |  |  |  |  |
|  | Mean | NA | 12.93 | 12.93 |  |
|  | SD | NA | 7.53 | 7.53 |  |
|  | N | 0 | 14 | 14 |  |
| Checking-in |  |  |  |  | 0.211^1^ |
|  | Mean | 4.00 | 2.00 | 3.33 |  |
|  | SD | 2.00 | 1.41 | 1.97 |  |
|  | N | 4 | 2 | 6 |  |
| Counseling |  |  |  |  | 0.747^1^ |
|  | Mean | 15.00 | 18.00 | 16.20 |  |
|  | SD | 7.07 | 13.88 | 9.72 |  |
|  | N | 6 | 4 | 10 |  |
| Overall Assessment |  |  |  |  | 0.707^1^ |
|  | Mean | 12.33 | 10.40 | 11.45 |  |
|  | SD | 5.35 | 6.39 | 5.63 |  |
|  | N | 6 | 5 | 11 |  |
| Prescription dispensing |  |  |  |  | 0.317^1^ |
|  | Mean | 5.00 | 11.00 | 8.00 |  |
|  | SD | NA | NA | 4.24 |  |
|  | N | 1 | 1 | 2 |  |
| Triage |  |  |  |  | 0.235^1^ |
|  | Mean | 8.33 | 5.80 | 6.38 |  |
|  | SD | 3.21 | 3.71 | 3.64 |  |
|  | N | 3 | 10 | 13 |  |

^1^Kruskal-Wallis rank sum test
