# Supplementary material for: Estimating the costs of adolescent HIV care visits and an intervention to facilitate transition to adult care in Kenya
Source: PLoS One. 2024 Feb 8;19(2):e0296734. doi: 10.1371/journal.pone.0296734 (PMC10852328; doi:10.1371/journal.pone.0296734)
Supplement: S7 Appendix — (DOCX) [file pone.0296734.s007.docx]

# S7 Appendix. Resources utilization by activity.

| Resource (unit) | Mean | SD | Resource (unit) | Mean | SD |
| --- | --- | --- | --- | --- | --- |
| **Blood draw in office** |  |  | **Overall Assessment** |  |  |
| Forms (count) | 2 | - | BP (min) | 7 | - |
| Gloves (count) | 1 | - | EMR (min) | 12 | 5.7 |
| Needle & Syringe (count) | 1 | - | Face mask (count) | 1 | - |
| **Booklet Review** |  |  | Forms (count) | 1.3 | 0.8 |
| ATP Booklet (count) | 1 | 0.0 | Gloves (count) | 2.2 | 1.1 |
| Forms (count) | 1.3 | 0.5 | Leaflets (count) | 1 | - |
| Pen (count) | 1 | 0.0 | Notebook (count) | 1 | - |
| **Prescription dispensing** |  |  | Pen (count) | 1 | 0.0 |
| EMR (min) | 5 | - | Pregnancy screening (count) | 1 | - |
| Forms (count) | 1.7 | 1.1 | Sputum container (count) | 1 | - |
| Notebook (count) | 1 | - | TB Screening (count) | 1 | - |
| Pen (count) | 1 | 0.0 | Thermometer (min) | 2 | - |
| Telephone time (min) | 1 | - | Tongue Depressor (count) | 2 | - |
| **Blood draw in laboratory** |  |  | **Counseling** |  |  |
| Forms (count) | 1.5 | 0.7 | EMR (min) | 10.8 | 4.9 |
| Gloves (count) | 2 | 0.0 | File (count) | 1 | - |
| Needle & Syringe (count) | 1 | 0.0 | Forms (count) | 1.8 | 1.2 |
| Vacutainer (count) | 1.5 | 0.7 | Leaflets (count) | 2 | - |
| **Checking-in** |  |  | Notebook (count) | 1 | 0.0 |
| BP (min) | 3 | - | Pen (count) | 1.25 | 0.4 |
| EMR (min) | 4 | 2.0 | Prop (count) | 3.5 | 0.7 |
| Forms (count) | 1.4 | 0.7 | Tablet (min) | 7 | - |
| Notebook (count) | 1 | 0.0 |  |  |  |
| Pen (count) | 1 | 0.0 |  |  |  |
| Scale (min) | 1 | - |  |  |  |
| **Triage** |  |  |  |  |  |
| BP (min) | 2.8 | 0.4 |  |  |  |
| EMR (min) | 4 | 1.0 |  |  |  |
| Forms (count) | 1 | 0.0 |  |  |  |
| Scale (min) | 1 | 0.0 |  |  |  |
| Stadiometer (min) | 1.1 | 0.3 |  |  |  |
| Thermometer (min) | 1.4 | 0.5 |  |  |  |
