# Supplementary material for: Estimating the costs of adolescent HIV care visits and an intervention to facilitate transition to adult care in Kenya
Source: PLoS One. 2024 Feb 8;19(2):e0296734. doi: 10.1371/journal.pone.0296734 (PMC10852328; doi:10.1371/journal.pone.0296734)
Supplement: S8 Appendix — (DOCX) [file pone.0296734.s008.docx]

# S8 Appendix. Unitary price for each resource included in the analysis.

| Activity | Resource | Category | Unitary Price (Ksh) | Unitary Price (USD) | Source |
| --- | --- | --- | --- | --- | --- |
| Blood draw in laboratory | Clinical Officer (min) | Human Resources | 10.36 | 0.10 | Field data collection |
| Blood draw in laboratory | Forms (count) | Office Supplies | 11.00 | 0.10 | Expert Consultation |
| Blood draw in laboratory | Gloves (count) | Disposable Materials | 17.50 | 0.16 | Field data collection |
| Blood draw in laboratory | Needle & Syringe (count) | Disposable Materials | 56.10 | 0.52 | Field data collection |
| Blood draw in laboratory | Vacutainer (count) | Disposable Materials | 36.98 | 0.34 | Field data collection |
| Blood draw in office | Clinical Officer (min) | Human Resources | 10.36 | 0.10 | Field data collection |
| Blood draw in office | Forms (count) | Office Supplies | 11.00 | 0.10 | Expert Consultation |
| Blood draw in office | Gloves (count) | Disposable Materials | 17.50 | 0.16 | Field data collection |
| Blood draw in office | Needle & Syringe (count) | Disposable Materials | 56.10 | 0.52 | Field data collection |
| Booking | EMR (min) | Information System | 1.08 | 0.01 | Expert Consultation |
| Booking | Nurse Counselor (min) | Human Resources | 10.36 | 0.10 | Field data collection |
| Booklet Review | ATP Booklet (count) | ATP Booklet | 132.62 | 1.23 | Field data collection |
| Booklet Review | Forms (count) | Office Supplies | 11.00 | 0.10 | Expert Consultation |
| Booklet Review | Nurse Counselor (min) | Human Resources | 10.36 | 0.10 | Field data collection |
| Booklet Review | Pen (count) | Office Supplies | 11.00 | 0.10 | Expert Consultation |
| Checking-in | BP (min) | Clinical Equipment | 0.00 | 0.00 | Field data collection |
| Checking-in | EMR (min) | Information System | 1.08 | 0.01 | Expert Consultation |
| Checking-in | Forms (count) | Office Supplies | 11.00 | 0.10 | Expert Consultation |
| Checking-in | Notebook (count) | Office Supplies | 100.00 | 0.93 | Expert Consultation |
| Checking-in | Nurse Counselor (min) | Human Resources | 10.36 | 0.10 | Field data collection |
| Checking-in | Pen (count) | Office Supplies | 11.00 | 0.10 | Expert Consultation |
| Checking-in | Scale (min) | Clinical Equipment | 0.01 | 0.00 | Field data collection |
| Counseling | Counselor (min) | Human Resources | 3.75 | 0.03 | Field data collection |
| Counseling | EMR (min) | Information System | 1.08 | 0.01 | Expert Consultation |
| Counseling | File (count) | Office Supplies | 11.00 | 0.10 | Expert Consultation |
| Counseling | Forms (count) | Office Supplies | 11.00 | 0.10 | Expert Consultation |
| Counseling | Leaflets (count) | Educational tools | 160.00 | 1.49 | Expert Consultation |
| Counseling | Notebook (count) | Office Supplies | 100.00 | 0.93 | Expert Consultation |
| Counseling | Pen (count) | Office Supplies | 11.00 | 0.10 | Expert Consultation |
| Counseling | Prop (count) | Office Supplies | 110.00 | 1.02 | Expert Consultation |
| Counseling | Tablet (min) | Communication equipment | 0.09 | 0.00 | Field data collection |
| Overall Assessment | BP (min) | Clinical Equipment | 0.00 | 0.00 | Field data collection |
| Overall Assessment | Clinical Officer (min) | Human Resources | 10.36 | 0.10 | Field data collection |
| Overall Assessment | EMR (min) | Information System | 1.08 | 0.01 | Expert Consultation |
| Overall Assessment | Face mask (count) | Disposable Materials | 110.00 | 1.02 | Expert Consultation |
| Overall Assessment | Forms (count) | Office Supplies | 11.00 | 0.10 | Expert Consultation |
| Overall Assessment | Gloves (count) | Disposable Materials | 17.50 | 0.16 | Field data collection |
| Overall Assessment | Leaflets (count) | Educational tools | 160.00 | 1.49 | Expert Consultation |
| Overall Assessment | Notebook (count) | Office Supplies | 100.00 | 0.93 | Expert Consultation |
| Overall Assessment | Pen (count) | Office Supplies | 11.00 | 0.10 | Expert Consultation |
| Overall Assessment | Pregnancy screening (count) | Disposable Materials | 370.00 | 3.44 | Internet search |
| Overall Assessment | Sputum container (count) | Disposable Materials | 36.98 | 0.34 | Field data collection |
| Overall Assessment | TB Screening (count) | Educational tools | 538.00 | 5.00 | Internet search |
| Overall Assessment | Thermometer (min) | Clinical Equipment | 0.01 | 0.00 | Field data collection |
| Overall Assessment | Tongue Depressor (count) | Disposable Materials | 11.82 | 0.11 | Field data collection |
| Prescription dispensing | Clinical Officer (min) | Human Resources | 10.36 | 0.10 | Field data collection |
| Prescription dispensing | EMR (min) | Information System | 1.08 | 0.01 | Expert Consultation |
| Prescription dispensing | Forms (count) | Office Supplies | 11.00 | 0.10 | Expert Consultation |
| Prescription dispensing | Notebook (count) | Office Supplies | 100.00 | 0.93 | Expert Consultation |
| Prescription dispensing | Pen (count) | Office Supplies | 11.00 | 0.10 | Expert Consultation |
| Prescription dispensing | Telephone time (min) | Communication equipment | 14.00 | 0.13 | Field data collection |
| Triage | BP (min) | Clinical Equipment | 0.00 | 0.00 | Field data collection |
| Triage | EMR (min) | Information System | 1.08 | 0.01 | Expert Consultation |
| Triage | Forms (count) | Office Supplies | 11.00 | 0.10 | Expert Consultation |
| Triage | Nurse Counselor (min) | Human Resources | 10.36 | 0.10 | Field data collection |
| Triage | Scale (min) | Clinical Equipment | 0.01 | 0.00 | Field data collection |
| Triage | Stadiometer (min) | Clinical Equipment | 0.03 | 0.00 | Field data collection |
| Triage | Thermometer (min) | Clinical Equipment | 0.01 | 0.00 | Field data collection |

*Abbreviations. min: per minute; count: per unit of resource; EMR: Electronic Medical Records System; ATP: Adolescents Transition Package; BP: Blood Pressure machine; TB: Tuberculosis; Ksh: Kenyan shilling; USD: United States Dollar; Exchange rate: 107.67 Ksh per USD(Central Bank of Kenya); Field data collection: Collected in facilities; Internet search: found on webpages displaying price information for Kenya; Expert Consultation: no specific information was found on the input so we supplement it by consulting with ATTACH program officers located in Kenya.*
