# Supplementary material for: Estimating the costs of adolescent HIV care visits and an intervention to facilitate transition to adult care in Kenya
Source: PLoS One. 2024 Feb 8;19(2):e0296734. doi: 10.1371/journal.pone.0296734 (PMC10852328; doi:10.1371/journal.pone.0296734)
Supplement: Attachment — Submitted filename: Review Commentsv2.docx [file pone.0296734.s010.docx]

September 8, 2023

Ref. Number: PONE-D-22-28780

Dear Dr. Brian C. Zanoni,

Thank you very much for the opportunity to revise and resubmit our manuscript, entitled “Estimating the costs of adolescent HIV care visits and an intervention to facilitate transition to adult care in Kenya.” We have revised our manuscript in response to the comments made by reviewer and editor. We are returning two copies of the revision, one in which the changes are marked (“marked”) and the other in which all changes have been accepted (“clean”).

We very much appreciate your consideration and look forward to hearing from you.

Sincerely,

Enrique Saldarriaga, PhD

(corresponding author)

Responses:

The authors thank the efforts made by the Editor to guarantee an appropriate review of our manuscript. Please find our answers in *italics* to all comments and requests for changes. We are also submitting the revised version in track changes and clean versions.

**Editor**

We have had difficult securing a second reviewer for your manuscript. To prevent any further delay in rendering a decision on your manuscript, I have decided to base this decision on reviewer #1 and my own review of your manuscript. Your paper was of interest and can provide a value to the existing literature. As you can see below, there were both strengths and weaknesses in your paper. There are important areas of your paper that require careful attention. From my own reading of your paper, I am in agreement with the reviewer that your paper will make an important contribution and that your paper will benefit from a thoughtful revision. Please revise your paper in accordance with the reviews. In addition to the comments by reviewer #1, particularly regarding the language/grammar, Tables/Figures, and methodology, please address the following comments:

***Response:*** *We thank the Editor for providing feedback to facilitate the processing of our manuscript.*

Methods:

1. Clarify the main study outcomes from the original clinical trial and this study. Page 4 says the main study outcomes are transition readiness, retention in care, and viral suppression. The authors should clarify that this was for the parent study.

***Response:*** *We have clarified this in the second paragraph of the Introduction, where we describe the findings from the parent trial as part of providing context for our costing analysis. In addition, we have added a sub-section called “ATP Intervention” in the Methods section, where we provide much more detail on the ATP intervention procedures.*

*Page 4 and 5,*

*“****ATP Intervention***

*The ATP toolkit consisted of two evidence-based interventions[16,20]. The first is a structured booklet, ‘Taking Charge,’ used to guide the discussion about transitioning to adult care. Booklet design was informed by the US-based ‘Got Transition’ and USAID tools[21,22] and adapted to the Kenyan setting in collaboration with stakeholders (young people, policy makers, community members, caregivers, and clinicians). The second intervention was the validated Namibia HIV Disclosure Intervention, a comic book ‘Why I take my medicine’, that uses a narrative about soldiers fighting a battle with an infection as a rationale to take ART[23]. In intervention sites, ATP was provided during regularly scheduled clinic visits, every three months for one year. All clinics utilized a readiness assessment tool to track individual transition readiness scores[16].*

*Healthcare providers at intervention clinics received training on ATP toolkit use and were provided continuous support on APT implementation during the first 6 months of the study. Nurses most frequently administered the ATP intervention, followed by counsellors and peer educators[17]. While all three cadre of HCWs are involved in adolescent care and were trained to deliver ATP[16]. Further, nurses have a broader scope of work while nurse counselors are more specialized in providing coping tools and guidance[24].”*

2. A description of the ATP would help readers better understand the intervention. Also a brief description of the training, timing and delivery of the ATP would assist in the interpretation. Also clarify if the ATP is delivered individually or to a group of adolescents.

***Response:*** *We agree. We have added the “ATP Intervention” in the Methods section for this purpose.*

Results:

3. Results – resources utilization per activity. The first 3 sentences should be in the methods not results section.

***Response:*** *We agree with the editor. We have substantially revised the Methods and Results section to better separate the information in each section as well as to improve clarity, grammar, and phrasing.*

4. Would report the estimated cost of the ATP per clinic per adolescent not by day (8.24 adolescents per day?) since this number can be quite variable.

***Response:*** *We apologize for the confusion. We present the estimated annual costs per clinic of providing ART transition care using the ATP toolkit, for which we used the clinic client volume. We have rewritten the ‘Cost estimates’ sub-section to improve clarity.*

*Page 11*

*“Using the reported client volume of 8.24 ALH per day, the annual cost of ART transition care with ATP was 98,963.2USD per year in each clinic.”*

5. Should also include the total cost of the ATP per adolescent per year. How many sessions per year is the ATP?

***Response:*** *The intervention lasted for one year at an expected rate of one visit every three months (Njuguna et al, The Lancet HIV, 2022). There are no specific guidelines for the standard of care. We used three visits a year to estimate the annual cost; as detailed in the first paragraph of “Cost estimates.”*

PAGE 5

*“In intervention sites, ATP was provided during regularly scheduled clinic visits, every three months for one year. All clinics utilized a readiness assessment tool to track individual transition readiness scores[16].”*

*Page 10*

*“Assuming 3 clinics visits per year, the annual cost of the intervention per client was 98.7USD. ART-continuation-only visits cost 19.5USD (95%CI 17.5, 24) per session.”*

*However, the focus of the study is to present the difference between the cost per session, which could then be used in economic analysis at varying specific frequencies depending on the context.*

6. What is the total cost of the ATP per adolescent? For example how much does it cost for an adolescent to complete the package and transition to adult care?

***Response:*** *Please see our answer to the previous comment.*

**Reviewer 1**

This is a timely discussion about transitioning from adolescent to adult models of HIV care using a US-based tool adapted for Kenyan adolescents. The study represents a low cost, less time intensive intervention that has the potential to be useful in many areas (when adapted appropriately); however, this study is currently limited to the need for additional discussion about the tool, further details about the settings that it has been trialed in, and reassessing grammar.

***Response:*** *We thank the reviewer for the time and effort in reviewing our manuscript and positive feedback. We agree with the reviewer’s assessment, and have now revised the paper to reflect the suggested edits.*

**METHODS:**

Overall, more information is needed. Please provide an overview of the ATP model. While there is a citation of the study methods previously cited, a brief description of ATP should be presented here as well. Also what types of providers are usually in these clinics. Figure 2 (which needs a title) provides inferences to this briefly, but isn't mentioned until the results section. Also, please described the difference between nurses, nurse counselors, and providers.

***Response:*** *We agree the intervention should be presented at greater detail. We have added the sub-section “ATP Intervention” to the Methods section. This section also includes our findings from the cluster randomized controlled trial regarding the health care workers who most frequently administered the intervention and a brief description of the differences between types of health care workers.*

*Pages 4 and 5*

*“ATP Intervention*

*The ATP toolkit consisted of two evidence-based interventions[16,20]. The first is a structured booklet, ‘Taking Charge,’ used to guide the discussion about transitioning to adult care. Booklet design was informed by the US-based ‘Got Transition’ and USAID tools[21,22] and adapted to the Kenyan setting in collaboration with stakeholders (young people, policy makers, community members, caregivers, and clinicians). The second intervention was the validated Namibia HIV Disclosure Intervention, a comic book ‘Why I take my medicine’, that uses a narrative about soldiers fighting a battle with an infection as a rationale to take ART[23]. In intervention sites, ATP was provided during regularly scheduled clinic visits, every three months for one year. All clinics utilized a readiness assessment tool to track individual transition readiness scores[16].*

*Healthcare providers at intervention clinics received training on ATP toolkit use and were provided continuous support on APT implementation during the first 6 months of the study. Nurses most frequently administered the ATP intervention, followed by counsellors and peer educators[17]. While all three cadre of HCWs are involved in adolescent care and were trained to deliver ATP[16]. Further, nurses have a broader scope of work while nurse counselors are more specialized in providing coping tools and guidance[24].”*

*In addition, we have expanded the information about the ATP in the Introduction.*

*Pages 3 and 4*

*“Given the urgent need to develop tools that aid health care workers (HCWs) to facilitate effective transition of children and adolescents to adult care, the Adolescent Transition To Adult Care for Adolescents Living with HIV in Kenya (ATTACH) study (National Institutes of Health, Grant Number 1R01HD089850-01) developed an adolescent transition package (ATP)[16], which incorporates evidence-based interventions to improve the readiness of young people, (15 to 24 years) with HIV to transition into HIV adult care. ATP effectiveness was evaluated in a cluster randomized clinical trial (RCT) across 20 clinics in four Kenyan counties, assigned 1:1 to either the ATP intervention or standard of care for one year. Results showed the scores of participants in ATP clinics were significantly higher for overall readiness (1.7, 95%CI 0.3, 3.1) and HIV literacy (1, 95%CI 0.2, 1.7) than those in the standard of care clinics[17].*

*We sought to estimate the total cost of an HIV care visit for adolescent transition, and the incremental cost of implementing the ATP package for care transition in Kenya. These costs can provide important evidence about the economic feasibility of the ATP intervention and inform policymakers in making resource allocation decisions. Our results can also inform future economic evaluations of HIV interventions.”*

Within the "Study Setting" there is mention of a "Review Booklet." What is this and who normally does this activity.

***Response:*** *We have added information about this in the sub-section “ATP Intervention”, Methods section (page 4 and 5; please see previous answer).*

The first sentence after Figure 1 should read as follows: "We developed a time and motion instrument to quantify how long HCWs SPENT..."

**Response:** This has been corrected. We thank the reviewer for the careful reading of our manuscript

There is also mention of "ATTACH budgetary." Please describe what this is and provide a citation for it.

**Response:** Thank you for pointing this out. We meant to say the “research project expense information.” This data is internal to the execution of the research project and allowed us to obtain estimates of unitary costs of the resources used to provide services. Supplemental Materials 5 contains an exhaustive list of the unitary costs estimated and the source used for each item.

Also provide a rationale for why the ATP costs were calculated in the manner that they were.

**Response:** We have now added a paragraph at the beginning of the Methods section providing rationale for the methodology we used.

*Page 4*

*“We conducted a time-driven activity-based, micro-costing analysis from the provider perspective to estimate the total cost of an adolescent clinic visit and the incremental costs of implementing the ATP[18]. We categorized each service into activities (e.g., patient’s overall health assessment, booklet review, viral load testing), and each activity into inputs (e.g., time of physician to conduct the health assessment, the booklet itself, laboratory materials).”*

The entire ANALYSIS section should be reviewed for grammar.

**Response:** We have substantially revised the Analysis section to improve grammar, readability, and overall clarity.

**RESULTS:**

What is "member of Operation Triple Zero."

**Response:** We initially used this term to provide some examples of how participants described their role. We have removed “operation triple zero” from the list because is less self-explanatory than the other ones and could be distracting for the readers.

Grammer should be checked within the section as well.

**Response:** We have revised the Results section to improve grammar, readability, and overall clarity.

In the subsection titled "Resource utilization per activity" (please note, I have removed the "S" after "Resources."), provide additional information about how internet searches for marker prices were obtained.

**Response:** We thank the reviewer for this comment. We realize this information is better suited in the Methods sections, under Data Collection. We have moved this information and provided more details to clarify our procedures.

*Page 6*

*“We used the ATTACH project expenses information obtained via field data collection to obtain the unit cost of resources utilized. When data on unit price was not available we searched for Kenya-specific information on websites offering medical supplies; if this information was not available we consulted with in country experts (program officers and research coordinators). We calculated the cost of developing and delivering the booklet, office supplies, and medical equipment. We estimated the unit cost of the ATP booklet by adding up costs used in development (content creation, illustration, and translation) and printing (delivery to the facility), divided by the average number of ALH seen per day, as reported by HCWs. Wages of physicians, nurses, and counselors were collected from administrative offices from participating facilities. Research costs were excluded to estimate the programmatic cost of ATP implementation in a clinic setting.”*

**TABLES:**

Table 1 needs a more descriptive title.

**Response:** Thank you for the suggestion, we have improved the Table’s title.

*Page 19*

*“Table 1. Respondents’ demographic and labor characteristics by site type.”*

In Table 2- please provide a footnote describing communication equipment. (This table would be easier to understand with a description of the ATP intervention in the methods section.)

**Response:** We have expanded the information provided in Table 2 and added a footnote for Table 3 as well.

*Page 20*

*“Notes: ^1^Includes electronic devices used for appointment management (i.e., scheduling, reminder, cancellation). ^2^Includes Electronic Medical Records system. See Supplemental material 6 for details on all resources included in each category.”*

**FIGURE:**

All figures need better resolution and most are blurry and the lightest gray font is challenging to read if someone prints the manuscript in black and white. All figures in this section need a title.

**Response:** We apologize for the inconvenience. Per the journals style requirements, we included the figures titles in the manuscript and uploaded the figures separately. However, this time we have included the figures’ title in the ‘description’ during the uploading process, so the caption will hopefully show next to the images in the submission document.

Regarding the figures’ definition. We agree it could be improved. We are submitting higher quality images.

Figure 2 (which I'm assuming is the one with "Control Facilities" as A and "Intervention facilities" as B but is currently unlabeled including no Figure number and no title) would benefit from a footnote box describing these roles.

**Response:** We have added a description of the roles in the sub-section “ATP Intervention”, Methods section.

*Page 5*

*“Healthcare providers at intervention clinics received training on ATP toolkit use and were provided continuous support on APT implementation during the first 6 months of the study. Nurses most frequently administered the ATP intervention, followed by counsellors and peer educators[17]. While all three cadre of HCWs are involved in adolescent care and were trained to deliver ATP[16]. Further, nurses have a broader scope of work while nurse counselors are more specialized in providing coping tools and guidance[24].”*

In Figure 4- there are several numbers in the smaller boxes within either of the 2 columns that need to be displayed differently as they overlap with other number.

**Response:** We agree. The new images also improve readability. However, please consider that the submission document stretches all images to a full page, when they are meant to be just a fraction of it, which affects its quality.
