# Supplementary material for: Estimating the costs of adolescent HIV care visits and an intervention to facilitate transition to adult care in Kenya
Source: PLoS One. 2024 Feb 8;19(2):e0296734. doi: 10.1371/journal.pone.0296734 (PMC10852328; doi:10.1371/journal.pone.0296734)
Supplement: Attachment — Submitted filename: Review Commentsv3.docx [file pone.0296734.s011.docx]

December 11, 2023

Ref. Number: PONE-D-22-28780

Dear Dr. Brian C. Zanoni,

Thank you very much for this second opportunity to revise and resubmit our manuscript, entitled “Estimating the costs of adolescent HIV care visits and an intervention to facilitate transition to adult care in Kenya.” We have revised our manuscript in response to the comments made by reviewers. We are returning two copies of the revision, one in which the changes are marked (“marked”) and the other in which all changes have been accepted (“clean”).

We very much appreciate your consideration and look forward to hearing from you.

Sincerely,

Enrique Saldarriaga, PhD

(corresponding author)

Responses:

**Reviewer #2:**

The study topic is relevant and the paper is generally well written. The authors have also comprehensively addressed the reviewer comments. I have noted a few issues that should be fixed before publication:

1. The authors should insert a space at the end of sentences and in-text citations; most of the references need this revision.

***Response:*** *We have now added an extra space before each of the references in the manuscript.*

2. The following sentence in the introduction:
Results showed the scores of participants in ATP clinics were significantly higher
for overall readiness (1.7, 95%CI 0.3, 3.1) and HIV literacy (1, 95%CI 0.2, 1.7) than those in the standard of care clinics[17].
Which measure of association is referenced above? The point estimate and the CI is given, but the measure is not named. Since the measure is documented very well in the referenced study, the authors should consider doing the same here.

***Response:*** *Thank you for pointing out this, it was an oversight. We have added the information to the mentioned sentence, it now reads:*

*“Results showed the scores of participants in ATP clinics were significantly higher for overall readiness (adjusted mean difference 1.7, 95%CI 0.3, 3.1) and HIV literacy (adjusted mean difference 1, 95%CI 0.2, 1.7) than those in the standard of care clinics [17].”*

3. Methods: in the second paragraph of the ATP intervention section, the sentence below seems incomplete:
While all three cadre of HCWs are involved in adolescent care and were trained to deliver ATP.

***Response:*** *We have now deleted the word “while” from this sentence.*

4. Results: Resource utilization per per activity section: Is there a reason why t-test is capitalized?

***Response:*** *This was a typo and we have made it lower case.*

5. Under the costs estimates in the results section: Was there a difference between ART continuation only visits and the usual care delivered to controls? Otherwise, what would explain the difference the cost for the latter (USD 19.5) and the control (USD 29.8)? Therefore, are we comparing the cost of an intervention and usual care (in this case USD 32.9 vs USD 19.5) or two interventions (USD32.9 vs USD29.8)? A clarification here would really be helpful.

***Response:*** *The main difference is that in both the control and intervention visits, providers offer services for ART-continuation and transition to adult HIV care. In addition, we estimated the cost of a visit focused solely on ART-continuation. We realize this was not sufficiently clear, so we have edited the last paragraph of the sub section “Analysis” from Methods.*

*Page 7:*

*“We also estimated the cost of a pediatric visit in which the provider does not offer transition to adult HIV care, that the visit is focused solely on ART-continuation. This estimate allowed us to quantify the additional cost associated with transition to adult care services in both control and intervention facilities. We obtained an overall estimate of the time spent in such visits from the HCWs interviews. Additionally, we conducted a scenario analysis assuming 5 years of useful life for the ATP booklets, with the volume of clients kept constant.”*

**Reviewer #3:**

Thanks for the opportunity to review this revised manuscript. I did not review the original, but have noted the revisions suggested by the previous reviewer and the editor.

This is an interesting manuscript costing the addition of an adolescent transition package (ATP) in Kenya compared to SOC services.

There are a few edits required prior to publication, listed below.
Abstract

- Add that in the randomised clinical trial, facilities were randomised 1:1 to the intervention and control arms

***Response:*** *We have now edited this sentence to read:*

*“Data was collected from 13 HIV clinics involved in a randomized clinical trial evaluating ATP in western Kenya.”*

*We decided not to mention 1:1 randomization as we only collected data in a subset of clinics (and an odd number) which may raise confusion about the methods. Additionally, the focus of the paper is the costing and the protocol has been previously published. Further, the 1:1 randomization is mentioned in the methods section.*

- In results section suggest deleting "with HIV" as this point has already been made

***Response:*** *We have now deleted “with HIV.”*

- There appears to be duplication of "healthcare worker time" descriptions which could be combined and summarized

***Response:*** *We have now re-written this section of the abstract.*

Introduction

- Add reference after 2nd sentence ending "children on ART".

***Response:*** *We have moved the reference to the end of the sentence.*

- "...from 37% to 95% depending on THE setting" Suggest adding "the"

***Response:*** *We have added “the” to this sentence.*

- Paragraph 2....delete comma before (15 to 24 years)

***Response:*** *We have deleted this comma.*

Methods

- First sentence suggest a comma after "time-driven"

***Response:*** *We have added a comma to this sentence.*

- In study setting 20 clinics are included however throughout the manuscript and abstract 13 clinics are included, please align

***Response:*** *Although* *20 clinics were involved in the main study, we sampled from a subset for the costing study. We now clarify this in the abstract and in the methods section:*

*Methods, page 5:*

*We conducted time and motion observation of clinic visits at a subset of study clinics. Clinic visits were divided into activities and we enumerated inputs needed to complete each activity. We assigned prices to each input enumerated [25]. Data collection was conducted in-person by 10 trained research assistants using REDCap in 13 clinics.*

*Abstract:*

*Data was collected from 13 HIV clinics involved in a randomized clinical trial evaluating ATP in western Kenya.*

- ATP intervention: Suggest "The ATP toolkit consists of" rather than consisted of

***Response:*** *We have now made this change.*

- Typo on page 5 2nd paragraph, 2nd line: APT instead of ATP

***Response:*** *We have corrected this error. Thank you for the careful reading of our manuscript.*

- Suggest deleting "while" and starting with "All three cadres of HCW......" add "cadres" instead of "cadre"

***Response:*** *We have removed “while” from this sentence.*

- The last sentence "Further, nurses have ......" seems a bit odd, suggest adding more context or deleting.
Results

***Response:*** *We have deleted this sentence.*

- There are a number of supplementary materials included, suggest including most relevant as part of the main paper tables/figures or more clearly describing relevant findings since it is a bit distracting to have to flip to supplementary material repeatedly

***Response:*** *We have included the 5 main figures and tables in the manuscript and we present the details of our costing methodology and additional results in the supplemental appendix. As is common with costing papers, there are a large number of supplemental tables. We have elected to leave them in the supplement but have numbered the tables for ease of review.*

- There is a large difference in skills for example nurse counsellors at implementation sites compared to controls. Was this purposeful and the reason that these clinics were chosen as implementation sites? This is fine but the context in the results is not clear. Similar with clinical officers. Are these doctors/nurse clinicians/clinical associates? please clarify?

***Response:*** *The skills of the personnel were not assessed by our study. The activities each professional performs in every site is independent of both our costing study and the parent trial and did not influence their selection in intervention or control, as this was decided randomly. All cadre of personnel received training to administer the ATP intervention and supervision to ensure quality control. Therefore, we do not think the quality of the intervention or quality of overall ART care would vary across randomly selected intervention and control sites.*

- The difference/significance between lab-based and office based blood draws is not clear to me-please elaborate on the significance of specifying the location of these blood draws. Bloods would need to be done regardless so it's not clear to me why the costs would be different

***Response:*** *The activity blood draw in laboratory requires the use of additional medical materials which entails costs that are unrelated to the ATP intervention and therefore are only noted as a minor point. Supplemental Table 7 contains a detailed list of the resources used in each activity. Because this is not a main driver in the difference in costs across sites, we have edited the second paragraph of the Discussion accordingly.*

*Page 12:*

*“While we found some differences across control and intervention sites, these were not significant and therefore we opted for the average across all to enhance the accuracy of the metric. Increasing the booklet useful life to 5 years led to a slight reduction of 0.5USD in the total cost per session in intervention sites. This implies that the main cost driver is the time spent by HCWs on the delivery of the booklet. The only other difference observed was the with blood-draw practices across sites (Table 3). In the context of a programmatic scale up of the ATP intervention, the booklet design and delivery is the most important additional cost.”*

- Prescription dispensing and triage are important activities but comparing the frequency in the control and intervention clinics also needs context.

***Response:*** *We did not intend to make inferences about the difference in activity reporting between intervention and control sites as our objective was to collect the most accurate estimates of time and resources spent in each activity. The differences we found are merely qualitative. We have edited the sub-section “Sample description” from Results to clarify.*

*Page 8:*

*“We found qualitative differences in cadre composition and activity reported across intervention and control sites. HCW roles varied across control and intervention clinics. A greater proportion of HCWs listed their role as counselor in control compared to intervention sites (42.1% vs 18.5%, respectively).Nurse counselor was more frequently listed in intervention clinics (63% vs 23% in control, respectively), as was clinical officers (15.8% vs. 7.4% in control, respectively). See Supplemental Table 2 for more details. We found less variability among reported activities across sites. Prescription dispensing was more frequently listed in control clinics (14.6% in control and 7.5% in intervention), while triage was similar across both sites (18.8% in control and 15% in intervention, respectively), as was counseling (27.1% in control and 26.2% in intervention, respectively). See Supplemental Table 3 for more details.”*

- Under cost estimates: 2nd last sentence, delete "s" from 3 clinics..."

***Response:*** *We have now deleted this extra “s”.*

- The annual cost of ART transition with ATP of just under 100K USD per clinic seems quite a large cost, even though this translates to and additional 3.1 USD/visit!

***Response:*** *We have now addressed this point in the results section to provide more accurate estimates of the incremental cost associated with the intervention.*

*Page 8:*

*“Using the reported client volume, we estimated the annual cost of providing ALH HIV-care at 71,679USD for an average control clinic, and 115,882USD. The incremental cost associated with the use of the ATP is 44,202USD per year and clinic.”*

Discussion
- 2nd sentence 1st paragraph: suggest adding "...... 3.1 USD extra per visit"

***Response:*** *We have altered that sentence to the following:*

*“Thus, adding the ATP implementation to improve the transition to adult care for children and adolescents in Kenya was costs 3.1USD per visit.”*

- 2nd paragraph, instead of "volume" of ALH, suggest "number"

***Response:*** *We have now changed this sentence. We have elected to say “volume” as it’s common in costing studies to incorporate the clinic volume into the costs of services.*

*“The estimated cost of the ATP booklet was dependent upon the reported volume of ALH at the clinic.”*

- In the discussion there is a lot of focus on the different activities between intervention and control sites, which seems to lack relevance, particularly the discussion between lab- and office based bloods. Please also see previous comment, but the relevance of this and why this should differ between intervention and control is not clear

***Response:*** *We added the discussion about blood draw to explain the only other difference in costs beside the booklet, but we do agree it was unnecessary detail. We have edited the second paragraph of the Discussion to address this.*

*Page 12:*

*“Across costs categories, the largest driver of the ATP intervention cost was the ATP booklet, which generated a difference of 2.8USD (90.3% of the total difference) between control and intervention sites. The estimated cost of the ATP booklet was dependent upon the reported volume of ALH at the clinic. While we found some differences across control and intervention sites, these were not significant and therefore we opted for the average across all to enhance the accuracy of the metric. Increasing the booklet useful life to 5 years led to a slight reduction of 0.5USD in the total cost per session in intervention sites. This implies that the main cost driver is the time spent by HCWs on the delivery of the booklet. The only other difference found was associated with blood-draw practices across sites. In the context of a programmatic scale up of the ATP intervention, the booklet design and delivery is the most important additional cost.”*

- As mentioned in the results, the cost of 100K USD per clinic to implement ATP is quite significant and should be discussed. It is expected and affordable within the routine health services?

***Response:*** *Although the economic cost of implementing the ATP intervention may be substantial at clinics with a large volume of ART care transition visits for adolescents, there is likely cost saving to the health system associated with retaining adolescents on ART. The affordability (or budget impact) of this intervention is not within the scope of this study, which does not provide a full economic evaluation. Future studies are needed to assess affordability and cost-effectiveness (value for money). Additionally, we present economic costs of ATP implementation. Now that the intervention has already been developed, these startup costs would likely not be incurred to clinics choosing to implement the transition intervention.*

*We have added the following to the discussion section:*

*Page 14:*

*“Finally, while we find that the incremental cost of adding the ATP intervention to a clinic visit is small, the costs increase with higher clinic volume and may be substantial. However, improving adolescent retention on ART may result in averted costs of to HIV-related hospitalizations. Future analyses are needed to evaluate affordability and cost-effectiveness. Additionally, the estimated economic costs include those associated with designing the intervention and may not be applicable to clinics that incorporate the tool as its currently designed.”*
